# Manipulation of Gut Microbiota as a Key Target for Crohn's Disease

**DOI:** 10.3389/fmed.2022.887044

**Published:** 2022-06-16

**Authors:** Reem Rashed, Rosica Valcheva, Levinus A. Dieleman

**Affiliations:** Centre of Excellence for Gastrointestinal Inflammation and Immunity Research, Department of Medicine, University of Alberta, Edmonton, AB, Canada

**Keywords:** Crohn's disease, IBD, intestinal microbiota, probiotics, prebiotics, diet, gut health

## Abstract

Crohn's disease (CD) is an inflammatory bowel disease (IBD) sub-type characterized by transmural chronic inflammation of the gastrointestinal tract. Research indicates a complex CD etiology involving genetic predisposition and immune dysregulation in response to environmental triggers. The chronic mucosal inflammation has been associated with a dysregulated state, or dysbiosis, of the gut microbiome (bacteria), mycobiome (fungi), virome (bacteriophages and viruses), and archeaome (archaea) further affecting the interkingdom syntrophic relationships and host metabolism. Microbiota dysbiosis in CD is largely described by an increase in facultative anaerobic pathobionts at the expense of strict anaerobic Firmicutes, such as *Faecalibacterium prausnitzii*. In the mycobiome, reduced fungal diversity and fungal-bacteria interactions, along with a significantly increased abundance of *Candida* spp. and a decrease in *Saccharomyces cerevisiae* are well documented. Virome analysis also indicates a significant decrease in phage diversity, but an overall increase in phages infecting bacterial groups associated with intestinal inflammation. Finally, an increase in methanogenic archaea such as *Methanosphaera stadtmanae* exhibits high immunogenic potential and is associated with CD etiology. Common anti-inflammatory medications used in CD management (amino-salicylates, immunomodulators, and biologics) could also directly or indirectly affect the gut microbiome in CD. Other medications often used concomitantly in IBD, such as antibiotics, antidepressants, oral contraceptives, opioids, and proton pump inhibitors, have shown to alter the gut microbiota and account for increased susceptibility to disease onset or worsening of disease progression. In contrast, some environmental modifications through alternative therapies including fecal microbiota transplant (FMT), diet and dietary supplements with prebiotics, probiotics, and synbiotics have shown potential protective effects by reversing microbiota dysbiosis or by directly promoting beneficial microbes, together with minimal long-term adverse effects. In this review, we discuss the different approaches to modulating the global consortium of bacteria, fungi, viruses, and archaea in patients with CD through therapies that include antibiotics, probiotics, prebiotics, synbiotics, personalized diets, and FMT. We hope to provide evidence to encourage clinicians and researchers to incorporate these therapies into CD treatment options, along with making them aware of the limitations of these therapies, and indicate where more research is needed.

## Introduction

Crohn's disease (CD) is a sub-type of an inflammatory bowel disease (IBD) characterized by transmural chronic inflammation of the gastrointestinal tract (1). Research indicates a complex CD etiology involving genetic predisposition and immune dysregulation in response to environmental triggers (1). This disease can affect any part of the intestine, but it is most commonly found in the terminal ileum and colon (2). CD disrupts the body's normal ability to digest and absorb food, including eliminating waste (3). The nature of the inflammation is usually segmental, asymmetrical, and transmural. Most patients present with an inflammatory phenotype at diagnosis, but develop complications over time including strictures, fistulas, or abscesses, which often lead to surgery. Surgery rates for CD have declined due to novel advances in medical therapy, but the 5-year risk of first major abdominal surgery in CD remains at 18% (4). Although biological therapy has significantly improved patient outcomes, this progressively destructive disease can lead to bowel damage and disability, including intestinal failure and short-bowel syndrome (5). Diarrhea, abdominal pain, weight loss, fever, nausea, and vomiting are only some of the symptoms that occur in a relapsing and remitting fashion for patients (1). Up to 47% of patients also experience extra-intestinal manifestations (EIMs) related to joints, skin, liver, biliary tract, and eyes (6, 7). Some of these EIMs are associated with disease course and severity, ultimately contributing to morbidity and mortality (8–10). The financial burden on health systems is related to the direct health care costs of IBD hospitalization, surgery, and medication, especially the rising use of biologics (5).

## The Gut Microbiota

Trillions of microbes comprising bacteria, fungi, viruses, eukaryotes, and archaea colonize the human gut microbiota. The microbiome serves many functions including playing a role in metabolism, immune and nervous system regulation, and colonization resistance (11, 12). Markedly, the gut microbiota has been implicated in the initiation and perpetuation of IBD. Infusion of luminal content into both mice models (13). and into the excluded ileum after a surgical diversion in patients with CD (14). triggers a rapid response in the mucosal immune system. This data suggests that commensal bacteria and dietary components can trigger the inflammatory response seen in CD (13, 14). The chronic intestinal inflammation and subsequent damage of the intestinal mucosa in IBD is associated with dysbiosis in the structure of the microbiota. Studies have confirmed that reduced diversity and dysbiosis of the gut microbiota are more pronounced in CD compared to ulcerative colitis (UC), a different sub-type of IBD (11, 15).

### Intestinal Microbiome in CD

**Figure 1:**
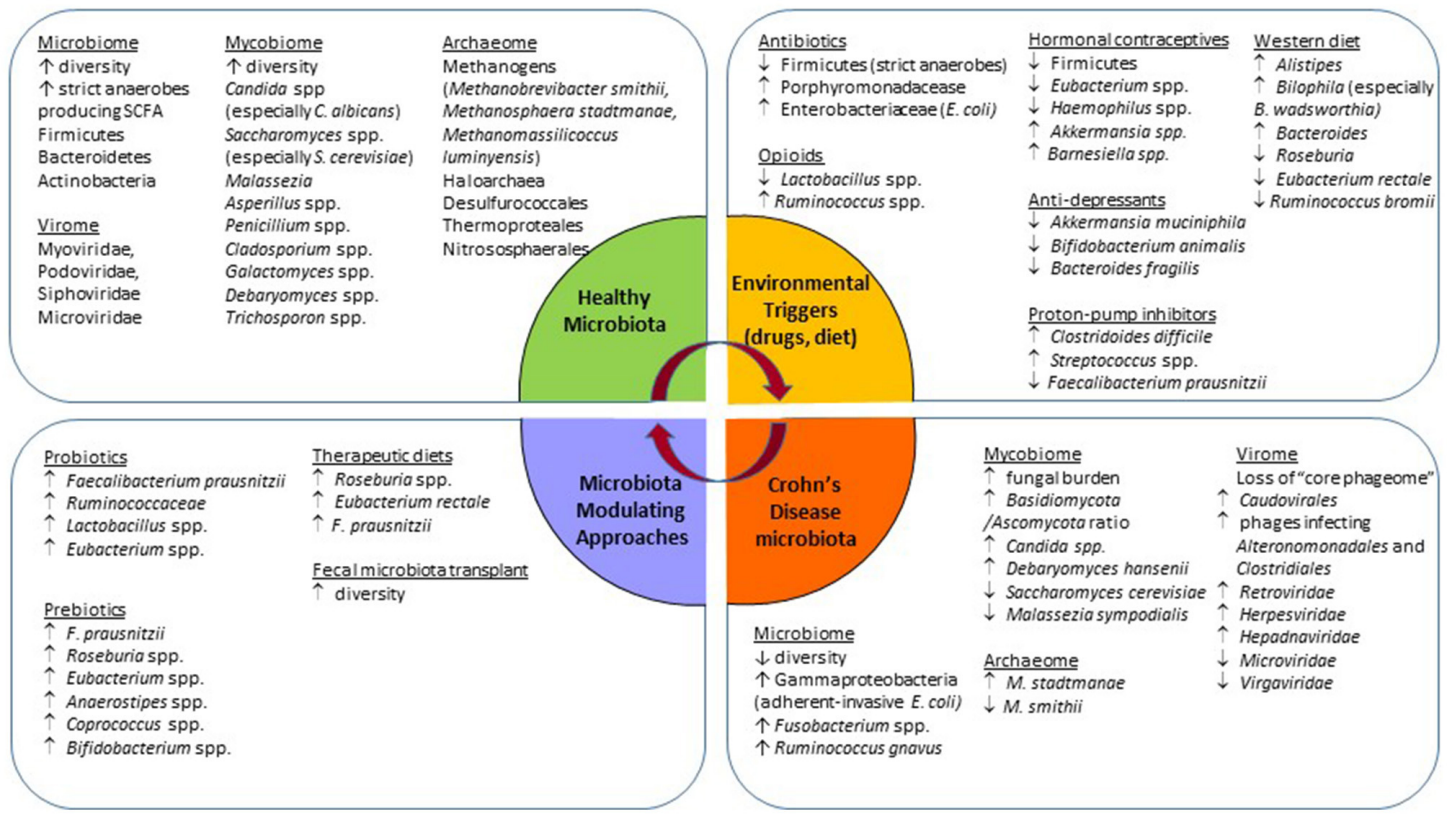
Schematic summary of observed gut microbiota changes associated with Crohn's disease, the usage of common medications, and approaches for microbiota modulation.

The gut microbiome comprising the intestinal bacteria is the most widely studied community. The healthy human commensal intestinal microbiota is composed of bacteria from three major phyla, namely Firmicutes, Bacteroidetes, and Actinobacteria (12). The bacterial signature of CD is reported to have a lower β-diversity (bacterial richness) with a decrease in obligate anaerobes, and an increase in facultative anaerobes (16) (Figure 1). This dysbiotic change facilitates the expansion of pathobionts that thrive in the presence of oxygen (16). A decrease in the relative abundance of Bacteroides and Firmicutes, especially Clostridiales butyrate-producing bacteria such as *Faecalibacterium prausnitzii* (17, 18). and *Roseburia* spp is seen in CD (19). The short-chain fatty acid (SCFA) butyrate, produced by these bacteria, acts as an energy substrate for colonocytes which further improves gut permeability by accelerating tight junction formation (12, 17). Butyrate also has anti-inflammatory effects such as inhibition of interleukin (IL)-6 release, lipopolysaccharide (LPS)-induced tumor necrosis factor-α (TNF-α) release, and suppression of the NF-κB inflammatory pathway via TNF-α activation (20). *F. prausnitzii* is a commensal bacterium with anti-inflammatory properties which reduces pro-inflammatory and increases anti-inflammatory cytokines (17). The relative lack of *F. prausnitzii* is associated with postoperative disease recurrence after ileocecal resection and re-anastomosis (18). CD is also characterized by an increased abundance of *Ruminococcus gnavus* and Gammaproteobacteria, such as *Escherichia coli*, which are mucosa-associated adherent-invasive bacteria. These pathogenic strains cross the mucosal barrier, adhere to and invade epithelial gut cells, and reproduce within macrophages to increase secretion of the pro-inflammatory cytokine TFN-α (11, 17, 21).

### Intestinal Mycobiome in CD

Although the mycobiome, or the fungal community, is composed of a small fraction of the microbiota (~10^5^ fungal cells per gram of fecal matters vs. 10^11^ bacterial cells per gram) (22), fungi play a myriad of roles related to host metabolism and host immunity in a broad range of ecosystems. This community is far more variable and dynamic than the bacterial community, responsive to environmental changes, and co-exists with the other microbial communities in the human body. Data suggests that there are three main fungal phyla in the gut, *Ascomycota, Basidiomycota*, and *Zygomycota*, and 10 “core” genera made up of *Candida* (especially *Candida albicans*), *Saccharomyces* (especially *Saccharomyces cerevisiae*), *Penicillium, Aspergillus, Cryptococcus, Malassezia* (especially *Malassezia restricta*), *Cladosporium, Galactomyces, Debaryomyces*, and *Trichosporon*. Importantly, as with bacteria, fungal communities have a spatial organization in the GI tract with luminal subset vs. mucosa-associated subset, the latter being more conservative and well-defined (23). Recently, it was shown that human mucosa-associated fungi, consisting mainly of the “immunoreactive” fungal genera *Candida* spp. and *Saccharomyces* spp. exert immunoprotective effects *via* upregulation of barrier function and transcription of epithelial genes involved in JAK/STAT signaling and DNA repair (23). Namely, in a healthy state the mucosa-associated mycobiota promoted barrier function through induction of CD4^+^ T helper cell-derived IL-22 and IL-17, resulting in protection against intestinal injury during antibiotic treatment and bacterial infection (23). Meanwhile, studies in patients with CD show an increased fungal burden in conjunction with an increase in abundance of the *Basidiomycota/Ascomycota* ratio, *C. albicans, C. tropicalis, Candida glabrata, Gibbrella moniliformis, Aspergillus clavatus, Alternaria brassicola, Cystofilobasidiaceace* family (24), and *Debaryomyces hansenii* (25) (Figure 1). *C. albicans*, a prevalent fungal species in the gut of patients with CD, is known as an inducer of T helper 17 (Th17) cells (26). Th17 cells are involved in immunity at the intestinal mucosal barrier. Under pathological conditions, such as IBD, Th17 secretes pro-inflammatory cytokines that aggravate inflammation (27), suggesting a possible pathobiont role of *C. albicans* in the case of IBD. Another study also implied the potential role of *Debaryomyces hansenii* in the perpetuation of chronic inflammation and tissue injury in CD (25). This fungus was found in abundance in patients with CD within the areas of surgical resections and inflamed regions of the intestines. Human isolates of *D. hansenii* effectively impaired colonic crypt repair *in vivo* illustrated by increased mucosal ulcerations and crypt loss (25).

Furthermore, a decrease in *S. cerevisiae* and *Malassezia sympodialis* is observed in CD (24). Sokol et al. demonstrated that *S. cerevisiae* significantly enhances the production of the anti-inflammatory cytokine interleukin (IL)-10 (24). While less is known about the role of *M. sympodialis* in intestinal inflammation, the genus *Malassezia* is often associated with skin disorders such as atopic eczema (28). Recent data suggest that *M. sympodialis* can stimulate mast cells to upregulate the release of cysteinyl leukotrienes and enhance IgE response, which results in a pro-inflammatory effect (29). More research is required to understand the exact role of *M. sympodialis* in CD.

Fungal-bacterial interactions were first recognized by Seelig in 1966 after antibiotic treatment in humans resulted in an overgrowth of *Candida* spp (30, 31). More recently, Sovran et al. demonstrated that antibiotic treatment significantly altered the fungal composition, confirming that bacterial dysbiosis could contribute to fungal dysbiosis (32). This study also showed that administration of *C. albicans* worsened disease severity, while administration of *Saccharomyces boulardii* reduced disease symptoms; however, both effects were lost after antibiotic treatment (32). It is hypothesized that the co-existence of fungi with specific intestinal bacteria is essential for the development/ amelioration of colitis. Sovran et al. exemplified this concept in a female C57BL/6J mice model where both *C. albicans* and *S. boulardii* required *Enterobacteriaceace* (modeled with colistin-resistant *E. coli* strains) to trigger their respective detrimental and beneficial effects on DSS-induced colitis (32). To support the role of interdependence between bacteria and fungi, gut fungal dysbiosis has been associated with reduced treatment response to fecal microbiota transplants (FMT) in recurrent *Clostridioides difficile* infections (CDi) (33). The microbiome of patients with CD is characterized by a decrease in fungal-bacterial interactions, as compared to healthy controls. A decreased abundance in *S. cerevisiae* is associated with a reduction in beneficial bacterial genera such as *Bifidobacterium, Blautia, Roseburia*, and *Ruminococcus*, whereas an elevation in *C. tropicalis* is positively associated with opportunistic bacteria such as *Serratia marcescens* and *E. coli* in CD (24).

### Intestinal Virome in CD

There is even less information about the role of virome in IBD as compared to the mycobiome, since little information is accessible through the public database, known as “viral dark matter” (34, 35). Gut virome is composed of eukaryotic and bacterial viruses (bacteriophages), including single-stranded DNA (ssDNA), double-stranded DNA (dsDNA), and RNA viruses. The intestinal virome is the most populated niche in the body, consisting of ~10^15^ bacteriophages, outnumbering commensal bacteria by a factor of 10 (36, 37). Although large and diverse, the intestinal virome is highly personalized and stable (38). The core “phageome” is mostly composed of dsDNA viruses most commonly from the *Caudovirales* order (*Myoviridae, Podoviridae*, and *Siphoviridae* families), and ssDNA viruses from the *Microviridae* family. More recently, a novel crAssphage and crAss-like phages have been identified as a common community in the virome and are associated with the bacterial phylum of *Bacteroidetes* (36, 38, 39).

Specific changes in the virome in patients with CD consist of a loss of the “core phageome” (Figure 1). Norman et al. describe a significant expansion of disease-specific *Caudovirales* bacteriophages and disease-and-cohort-specific changes in the virome of patients with CD and UC (40). CD-associated phageome dynamic changes are characterized by an increase in phages infecting *Alteronomonadales* and *Clostridiales* bacterial orders (41), and an inverse shift in *Caudovirales* vs. *Microviridae* bacteriophages (35). As for the eukaryotic virome, an increase in the abundance of *Retroviridae, Herpesviridae, Hepadnaviridae, Hepeviridae* families, and a decrease in the *Virgaviridae* family were observed (36, 37).

Although the exact role of the virome in IBD is not entirely clear, some evidence suggests that it may contribute to intestinal inflammation. Bacteriophages are important to bacteria as they drive bacterial diversity and fitness in the gut (42). They are involved in the horizontal transfer of genetic information between bacteria, including material related to pathogenesis and antibiotic resistance (42–44). In CD, there is an increase in the abundance of temperate phages which shift from lysogenic to lytic replication (43). The normal process of viral reproduction occurs *via* the lysogenic cycle, which involves the fusion of the nucleic acid of a bacteriophage and the host cell leading to proliferation (45). In contrast, the lytic cycle involves the penetration of a cell membrane, nucleic acid synthesis, and lysis of the host cell (37, 43). Lysis of gut bacterial hosts is theorized to release proteins, lipids, pathogen-associated molecular patterns, and antigens that trigger inflammatory pathways leading to pro-inflammatory cytokine induction and tissue damage (40). Additionally, *in vitro* studies have demonstrated that bacteriophages can stimulate macrophages to induce MyD88-dependent pro-inflammatory cytokine production, suggesting its role in innate immunity (46). Bacteriophages may also play a therapeutic role in CD treatment. A cocktail of three bacteriophages was demonstrated to reduce symptoms and significantly reduce fecal adhesive-invasive *E. coli* (AIEC) in DSS-induced colitis in mice. AEIC colonization of ileal mucosa in CD was correlated with disease activity and location, as well as postoperative recurrence (47). This bacteriophage cocktail may thus have therapeutic promise for patients with CD.

### Intestinal Archeaome in CD

Archaea are a domain of prokaryotic, single-cell organisms, collectively known as the archeaome (48). The knowledge of the human gut archeaome is limited and is mostly based on methodological concepts biased toward commensal bacteria. The majority of detected archaea in the gut are methane-producing organisms known as methanogens. Methanogens respire H_2_ and produce methane gas under anaerobic conditions. They exist in a syntrophic relationship with bacteria; by removing H_2_, methanogens improve bacterial fermentation efficiency in the gut and allow complete anaerobic degradation of organic material. Methanogens make up ~10% of gut anaerobes, as *Methanobrevibacter smithii* is the most common (49–51). Other common species detected in the gut are *Methanosphaera stadtmanae, Methanomassilicoccus luminyensis* (49, 52), as well occasionally several non-methanogenic strains, such as D*esulfurococcales, Sulfolobales, Thermoproteales, Nitrososphaerales*, and *Halobacteriales* (53–55). *M. smithii* has low immunogenic potential, suggesting its commensal role as a gut microbe. *M. luminyensis* plays a beneficial role through the degradation of trimethylamine (TMA) and trimethylamine-N-oxide (TMAO), both byproducts of choline microbial metabolism strongly associated with endothelial dysfunction and increased risk of cardiovascular disease (56). Lastly, *M. stadtmanae* has shown high immunogenic potential (57) and is suggested to be present in high abundance in pathological conditions (58, 59).

Changes in the archeaome related to CD show at least a 3-fold increase in *M. stadtmanae* and a reduction in *M. smithii*, as compared to healthy controls (Figure 1). Interestingly, these numbers normalize with IBD remission (49). The “syntrophic imbalance hypothesis” suggests that butyric acid, an SCFA in the gut, is an essential component for the regulation of archaea/bacteria biofilms in the gut. This hypothesis states that dysbiosis is a product of archaeal overgrowth and increased SCFA removal from intestinal biofilms, which in turn triggers bacteria to become endoparasitic and enter intestinal epithelial tissues, initiating and perpetuating chronic inflammation in the gut (60). More research is needed to support the proposed hypothesis, but recall that SCFAs, such as butyric acid, are reduced in patients with IBD, therefore promoting that butyrate-producing bacterial growth to improve intestinal barrier integrity should be a promising target of therapy (61, 62).

## Conventional Therapies in CD and Their Modulating Effects on Gut Microbiota

The goal of CD therapy is to achieve deep remission, that is, to induce and maintain symptomatic and endoscopic remission and mucosal healing. Common anti-inflammatory medications used in CD management include amino-salicylates, immunomodulators, corticosteroids, and biologics (1, 63, 64). Amino-salicylates are less used in CD due to their lack of efficacy in inducing/maintaining remission and preventing postoperative recurrence. Corticosteroids are used mainly as induction of remission medication but not for maintaining remission, also due to serious side-effects such as osteoporosis, diabetes, hypertension, and increased risk for infections. Immunomodulators are commonly used but also have side effects such as an increased risk for malignancies (such as lymphoma, non-melanoma skin cancers, myeloid disorders, and urinary tract cancers). Anti-tumor necrosis factor (TNF) biologics such as infliximab, adalimumab, and certolizumab, are generally well-tolerated, however; they are expensive and may increase the risk of infections as well as melanoma skin cancer. Vedolizumab (anti-integrin α4β7) and ustekinumab (anti-IL 12/23) are newer biological drugs with improved safety and efficacy profiles but lack long-term data (1, 2).

The efficacy of conventional therapy can be affected by certain factors related to the gut microbiota. For example, patients with IBD who achieve ’early' clinical remission at 14 weeks with anti-cytokine therapy (anti-TNF, anti-IL 12/23) have significantly higher microbial species richness at baseline compared to non-responders (65). The same study also found that nine microbial species at baseline were associated with early clinical remission in patients treated with anti-TNF and that three microbial species were related to response to anti-integrin therapy. Of those species, *Phascolarctobacterium faecium, Agathobaculum butyriciproducens*, and *Clostridium citroneae* were associated with increases in fecal SCFA production to produce anti-inflammatory effects. The decrease in inflammation by anti-TNF therapy is associated with modulation of the gut microbiome toward eubiosis. The microbiome of patients successfully treated with anti-TNF therapy slowly resembles that of healthy individuals. Studies demonstrated a decrease in Enterobacteriaceae (*E. coli* in particular) and *Ruminococcus*, with an increase in abundance of Bacteroidetes and Firmicutes (66).

## Concomitant Medications Used in CD and Their Modulatory Effects on Gut Microbiota

Recent studies have revealed that many commonly used drugs other than antibiotics also exhibit profound effects on the gut microbiota composition and function (Figure 1). An extensive review of the microbiota-modulation effect of non-antibiotic medications and the potential clinical consequences can be found elsewhere (67). Here, we summarize data with respect to drugs commonly used by patients with CD and their potential contribution to the observed intestinal dysbiosis, as well as disease onset and progression.

### Antibiotics

Antibiotic exposure has been identified as an environmental stressor contributing to the pathogenesis of IBD. In healthy humans, antibiotic use has demonstrated perturbations and a decrease in colonization resistance of the gut microbiota (68). Broad-spectrum antibiotics can affect the composition of at least 30% of gut microbes to cause a drastic shift in richness, diversity, and evenness (69, 70). Repeated exposure to antibiotics leads to a reduction in diversity, resulting in pathogenic overgrowth and dysbiosis (69, 70), increasing the risk of infection (71). Another mechanism in which antibiotics increase the risk of intestinal infections is related to their role in the thinning of the mucosal layer leading to barrier dysfunction (72). Various studies have found a significant association between prior antibiotic use and the development of CD (73, 74). Both exposures in the first 5 years of life (75) and 2–5 years prior to diagnosis (76). contribute to an increased risk of developing IBD. The association between antibiotic use and CD has been supported through metagenomic analyses, showing a decrease in the abundance of Firmicutes (such as *C. leptum*) and an expansion of gram-negative bacteria such as Porphyromonadacease, and Enterobacteriaceae (*E. coli*) (77–79).

### Hormonal Contraception

The use of oral contraceptive pills (OCP) is associated with a 30% increased risk for the development of IBD in a genetically susceptible host. In particular, there is a 24% higher risk of CD development in those who are exposed to OCP vs. those who are not (80). As OCPs function by using estrogen receptors, van Langen et al. observed that reduced estrogen receptor-β's (ER-β) mRNA expression, the most abundant estrogen receptor, and increased gut permeability preceded the onset of colitis in two animal models. Furthermore, the study found reduced ER-β mRNA levels in colonic biopsies from patients with IBD in relapse. Finally, *in vitro* experiments demonstrated an association between ER-β signaling and epithelial barrier function. They concluded that ER-β signaling has a role in maintaining epithelial barrier function, which in turn is related to IBD risk (81).

Mihajlovic et al. demonstrated that the use of combined hormonal contraceptives (CHC), consisting of natural or synthetic 17-β-estradiol and progesterone, was associated with significantly lower gut microbial diversity and richness due to CHC-induced decrease in endogenous estradiol and progesterone. These authors also identified bacterial groups such as unclassified Firmicutes, *Eubacterium* spp, and *Haemophilus* spp. that were less abundant in the CHC group, while *Akkermansia* and *Barnesiella* were enriched in the CHC group in comparison to healthy controls not using CHC. These data suggest that CHC-induced hormonal changes may affect gut microbiota diversity (82). Lastly, not only are OCPs associated with increased risk for CD but long-term use of OCPs in patients with established CD is also associated with an increased likelihood of surgery and risk of relapse (83). These recent data raise the need for a re-evaluation of the benefits vs. risk of OCPs not only in individuals with established CD but also in those at increased risk for CD, such as their first-degree relatives.

### Opioids

Opioids are the most common analgesics prescribed for pain management in IBD. In a study assessing the effect of hydromorphone in both DSS-induced colitis and spontaneous colitis (IL-10 knockout) mouse models of IBD, hydromorphone independently induced barrier dysfunction, bacterial translocation, disruption of tight junction organization, and increased intestinal and systemic inflammation. This effect was exacerbated with significant microbial dysbiosis in mice receiving hydromorphone in combination with DSS. These data warn against the use of opioids and that clinicians should opt for other methods of pain management in IBD as opioids can accelerate disease progression by dysregulation of the gut microbiota, leading to expansion of pathogenic bacteria, followed by their translocation, leading to worsening immune dysregulation and sustained chronic intestinal inflammation (84). Despite these adverse effects, a recent systematic review and meta-analysis found that 21% of outpatients with IBD and 62% of hospitalized patients with IBD use opioids for pain management. Opioid use was associated with female sex, depression, substance abuse, prior gastrointestinal surgery, biologic use, steroid use, along with a more severe disease course in IBD and increased healthcare use (85). Male C57Bl/6J mice subjected to intermittent morphine treatment display a significant decrease in the relative abundance of *Lactobacillus* spp. and an increase in *Ruminococcus* spp (86). In contrast, mice exposed to sustained morphine treatment show a significant increase in abundance in *Clostridium* spp. and *Rikenellaceace* family (86). The depletion of the gut microbiota in these mice with antibiotic treatment reduced analgesic potency following intermittent morphine treatment (86). These data suggest that an alteration in the gut microbiome following antibiotic therapy or intermittent opioid treatment is detrimental to inflammation (86). Human data on microbiota modulation by opioids is currently not available, hence it is not known if observations made in colitis models can be fully translational in patients with CD.

### Anti-depressants

It is well documented that up to 30% of patients with IBD are more likely to experience depression and that depression worsens IBD prognosis. A study assessing six types of antidepressants (phenelzine, venlafaxine, desipramine, bupropion, aripiprazole, and (S)-citalopram) for their microbial activity against 12 commensal bacterial strains revealed that certain antidepressive medications inhibit the growth of beneficial bacteria such as *Akkermansia muciniphila, Bifidobacterium animalis*, and *Bacteroides fragilis*. There was a significant reduction in bacterial viability, at a 5 logs cycle reduction (87). These findings demonstrated that certain antidepressants exhibit a strong antimicrobial effect against specific commensal gut bacteria (87), which is often overlooked. Although depression is associated with the onset of CD and UC, another study found that using certain treatments for depression, such as selective serotonin reuptake inhibitor (SSRI) and tricyclic antidepressant (TCA), was associated with disease reduction in CD (88). Therefore, while depression increases the risk of CD development, this risk may be altered by use of specific antidepressants (88). A bi-directional brain-gut axis interaction in patients with IBD refers to psychological disorders such as anxiety and depression and IBD activity. Patients with normal anxiety scores at baseline with active disease were almost 6 times more likely to develop abnormal anxiety scores during follow-up. In contrast, those who had the inactive disease at baseline, but had abnormal anxiety scores, had 2-fold higher rates of the flare of disease activity or need for glucocorticoids and escalation of therapy (89).

### Proton Pump Inhibitors

Proton pump inhibitors (PPIs) reduce gastric acid secretion and are widely used in upper GI disorders management, such as peptic ulcer disease, gastroesophageal reflux disease, and non-ulcer dyspepsia (90). Although PPIs exhibit a sound safety profile and have demonstrated significant benefits in acid-related gastrointestinal disorders, there are controversial data regarding their safety in IBD. In an observational analysis of three cohorts made up of 6,40,000 subjects, long-term PPI use was associated with an increased risk of developing IBD (91). This association was also found in multiple other studies correlating PPI use to increased risk of enteric infections such as *Clostridoides difficile* and IBD. A recent metagenomic analysis of three population cohorts (general, IBD, and IBS) also identified that PPI accounted for the largest number of drug-associated microbiota shifts, with a total of 40 altered taxa and 166 altered microbial pathways (92). The mechanisms behind this association are unclear but may be attributed to the reduction in gastric pH, thus introducing oral bacteria and promoting the growth of potentially pathogenic bacteria (as *Streptococcus*) (92, 93) and direct inhibition of certain commensal gut bacteria such as *Faecalibacterium* (92, 93). This leads to a weakening of barrier function and reduction of microbial diversity ultimately leading to dysbiosis (93). This collective data suggest that although PPIs have had profound effects on certain gastrointestinal diseases, they should not be prescribed beyond the minimal dose to induce benefit in patients.

## Methods of Modulating the Gut Microbiota in Crohn's Disease

Modulation of the gut microbiota is often used as an adjuvant therapy to conventional medication in IBD. Projects such as the Human Microbiome Project (94) and the European Metagenomics of the Human Intestinal Tract (MetaHIT) (95) have had profound contributions to the identification and characterization of the microbes that distinguish health and disease states. Animal models have also been instrumental in the study of the microbiome and IBD. Hernández-Chirlaque et al. (96), confirm that the absence of a microbiome in germ-free (GF) and conventional mice treated with antibiotics cocktail [“pseudo-GF”] experience reduced DSS colonic inflammation but also have impaired barrier function. This data suggests that enteric bacteria are essential for the development of DSS-induced colitis and epithelial barrier function (96). Germ-free mice studies also link the commensal microbiota to the promotion of intestinal immunity through mechanisms such as toll-like receptor (TLR) expression (97). antigen-presenting cells, lymphoid follicles, CD4^+^ T cells (98, 99), and antibody expression (100). Twin studies demonstrate that despite the heritability of the gut microbiome, environmental factors related to diet, drugs, and lifestyle can be larger determinants of microbiota composition and disease development (101, 102). This data suggests that enteric bacteria are essential for the development of chronic intestinal inflammation. A logical development then is to attempt to modify the intestinal microbiome (not only its composition but also the microbial metabolic fitness) in order to sustain or even reverse pathophysiological changes observed in CD.

### Probiotics in CD

The word probiotic originates from the Latin “pro” and Greek “bios” which translates to “for life” (103). The currently accepted definition is “live microorganisms that, when administered in adequate amounts, confer a health benefit on the host” (104). This definition encompasses all microbes, although the bacteria of gut microbiota is the most widely studied. The most common probiotic cocktail with proven efficacy in UC and chronic pouchitis is Visbiome^®^ (formerly VSL#3) (105, 106). Visbiome contains eight different bacterial strains from well-known probiotic species *Lactobacillus plantarum* DSM24730, *Streptococcus thermophilus* DSM24731, *Bifidobacterium breve* DSM24732*, L. paracasei* DSM24733, *L. delbrueckii subsp. bulgaricus* DSM24734, *L. acidophilus* DSM24735, *B. longum* DSM24736, and *B. infantis* DSM24737 (107). In particular, bacteria such as *Lactobacilli* and *Bifidobacteria* have been extensively tested for anti-inflammatory effects in colitis and their beneficial effects on gut motility, particularly for the treatment of constipation (108). VSL#3 is not yet shown to be effective in patients with CD, as compared to those with UC (109, 110).

However, Visbiome started within 30 days after ileocecal resection followed by re-anastomosis prevented disease recurrence in a study with 120 patients with CD, suggesting that the timing of this probiotic cocktail after resection is important. However, more studies are needed within this scope (111). *In vitro* supplementation with commensal strains such as *F. prausnitzii* seems to show favorable results (18, 112). Some successes have been demonstrated with the administration of *S. boulardii* in combination with mesalazine in patients with CD, where a significant reduction in the incidence of relapse occurred as opposed to the mesalazine only-treated group, however, this was a small study (113). Although there are few studies assessing the effect of *S. boulardii* in CD, the existing evidence only slightly favors the use of the yeasts as a probiotic in CD in certain populations (such as non-smokers) (114). Table 1 summarizes various probiotics and their clinical efficacy in CD. Apart from the use of *Saccharomyces boulardii* as a probiotic, there is no evidence that sufficiently supports the use of the current probiotics to induce clinical remission in CD. More large, high-quality well-powered studies are needed to determine the specific factors needed for the efficacy of probiotics in CD.

**Table 1:** Use of probiotics in patients with Crohn's disease.

**Type of study**	**Participants (*n*)**	**Duration** **(months)**	**Intervention**	**Control**	**Reference**	**Outcome**
**Probiotics to induce clinical remission**						
Open-label	17	0.5	*Saccharomyces boulardii*	None	(115)	Modest symptomatic improvement
RCT	11	6	*Lactobacillus rhamnosus* GG + Corticosteroids	Placebo + Cortico- steroids	(116)	No benefit
Open-label	10	13	*Bifidobacterium longum, B. breve, Lactobacillus casei* + Plantago ovata	None	(117)	Symptomatic improvement
RCT	35	6	*B. longum* + FOS/inulin	Placebo	(118)	Symptomatic improvement
**Probiotics to maintain clinical remission**						
RCT	17	3	*S. boulardii*	Placebo	(115)	Improvement
RCT	28	12	*E. coli Nissle 1917*	Placebo	(119)	No benefit
RCT	35	6	*S. boulardii*	Pentasa	(113)	Prevented relapse
RCT	11	6	LGG	Placebo	(116)	No benefit
RCT	75	42	LGG	Inulin	(120)	Deterioration (NS)
RCT	30	12	VSL #3	Placebo	(109)	Deterioration (NS)
	30	12	*S. boulardii*	Placebo	(121)	No benefit overall; favorable in non-smokers?
RCT	62	1	Symprove (*L. rhamnosus, L. plantarum, L. acidophilus, Enterococcus faecium*)	Placebo	(122)	No Benefit
Observational	200	-	Various	-	(123)	Reduced adverse events
**Probiotics to prevent post-operative recurrence**						
RCT	40	12	Rifaximin – VSL #3	Mesalamine	(124)	Lower incidence of endoscopic recurrence
RCT	45	12	LGG	Placebo	(125)	Deterioration (NS)
RCT	98	6	*Lactobacillus johnsonii*	Placebo	(126)	No benefit
RCT	30	24	“Synbiotic 2000”	Placebo	(127)	No benefit
RCT	70	3	*L. johnsonii*	Placebo	(128)	No benefit
RCT	120	12	VSL #3	Placebo	(109)	No statistically significant benefit

*S. boulardii, Saccharomyces boulardii; LGG, Lactobacillus rhamnosus GG; FOS, fructo-oligosaccharide; NS; not significant; RCT, randomized controlled trial*.

### Prebiotics in CD

Prebiotics are defined as “a substrate that is selectively utilized by host microorganisms conferring a health benefit (129)”. These health benefits are not necessarily limited to the colon, but also occur in the oral cavity, urogenital tract, lungs and skin (130). Typically, prebiotics were thought to be limited to non-digestible carbohydrate sources, such as fructooligosaccharides, galacto-oligosaccharides, resistant starches, pectin, arabinoxylan, and whole-grains, but an updated definition now includes non-carbohydrate sources, such as polyphenols and certain lipids (130). The most extensively tested type of prebiotic is inulin-type β-fructans, which are naturally derived from food sources such as chicory root, and onions (111, 129).

Prebiotics confer benefit to the host through their fermentation by some commensal microbes in the gut resulting in compositional and metabolic modulations/alterations (111, 131–133). Prebiotics are a non-selective growth substrate, allowing the simultaneous growth of multiple beneficial strains, such as *F. prausnitzii, Roseburia* spp., *Eubacterium* spp., *Anaerostipes* spp., *Coprococcus* spp., *Bifidobacterium* spp (134). Furthermore, prebiotics is synergistically co-metabolized by several distinct microbial groups such as butyrate-producing *F. prausnitzii* and acetate-producing *B. adolescents* leading to more efficient co-fermentation (135). However, it is important to note that not all non-digestible carbohydrates can be considered prebiotics. For example, feeding IL-10 knockout mice with dextrin fibers derived from corn resulted in microbiota shifts such as an increase of some Bacteroidetes families (*Porphyromonadaceae* and *Prevotellacea*) vs. reduction of strict anaerobic Firmicutes (*Incertae Sedis XIV, Lachnospiraceae, Ruminococcaceae*, and *Lactobacillaceae*). These changes were also seen in conjunction with reduced pro-inflammatory pathways such as IL12p70, IL-6, and chemokine ligand 1 (CXCL) (136). Despite these differences, these mice did not experience any improvement in colonic inflammation—or in other words, no health benefits to the host (136).

Arguably, a major function of prebiotics is their fermentation by commensal microbes into SCFAs. Propionate, acetate, and butyrate are the main SCFAs (20, 137, 138). SCFAs enhance mucus secretion, increase anti-microbial peptides, lower the pH of the colon to decrease oxygen levels, and inhibit the growth of pathogenic anaerobes. SCFAs also upregulate the expression of tight junction proteins to maintain a healthy functional immune system and intestinal barrier (137, 139) and reduce the production of putrefactive substances, such as ammonia, indole, branch-chain fatty acids, and phenol (137). Butyrate acts as the main energy source for colonocytes. This SCFA is of particular interest in IBD, as it is significantly reduced in colonic cells leading to autophagy and energy deprivation (140). Butyrate inhibits NF-κB activation *via* an increase in cytoplasmic inhibit (IKB) thus inhibiting pro-inflammatory cytokines and chemokines, such as interferon-γ (INF-γ), pro-inflammatory chemokine CXCL-8 (IL-8) in Caco-2 cells, and TNF-α (20, 141–143).

In a well-powered placebo-controlled study, Benjamin et al. found that supplementing 120 patients with active CD with 15g/day of inulin-type β-fructans had no clinical benefit (144). However, De Preter et al. and Joossens et al. using 10 g twice daily oligofructose-enriched inulin (OF-IN) in 67 inactive or mild to moderately active CD reported microbiota shifts toward an increase in *B. longum* that positively correlated with an improvement in CD disease activity and that OF-IN intake increased fecal butyrate and acetaldehyde (145, 146). As summarized in Table 2, few CD studies with prebiotics have demonstrated inconsistent results, likely due to a dose-dependent effect of prebiotics, disease stage/severity, and adverse effects, such as bloating, which may mask symptom improvement; therefore, more RCT studies with adequate power assessing objective disease parameters in association with protective mechanisms are needed to discern their effect in various CD phenotypes.

**Table 2:** Use of prebiotics to induce or maintain clinical remission in patients with Crohn's disease.

**Type of study**	**Participants (*n*)**	**Duration** **(months)**	**Intervention**	**Control**	**Outcome**	**Reference**
RCT	41	1	FOS, 15g/d	Maltodextrin, 15g/d	No benefit	(144)
Open label	22	4	Two 8-ounce cans/day of IBDNF	None	Significant decrease in plasma phospholipid levels of arachidonic acid with an increase in eicosapentaenoic acid and docosahexaenoic acid.	(147)
RCT	67	1	OF-IN, 20g	Placebo	Improvement in disease activity associated with increase in *Bifidobacterium longum*, and butyrate	(145, 146)
Observational case-control	303	-	None	None	Patients with active CD presented lower fructan and lower oligofructose intakes than inactive CD or control groups. Negative correlation between HBI wellbeing score and fructan and oligofructose intakes.	(148)

*RCT, randomized control trial; FOS, fructo-oligosaccharide; IBDNF, Inflammatory Bowel Disease nutrition formula; OF-IN, oligofructose-enriched inulin; CD, Crohn's disease, HBI, Harvey-Bradshaw Index*.

### Synbiotics in CD

Synbiotics are a combination of carefully curated prebiotics and probiotics that work together to exert a synergistic effect. While prebiotics encourages the proliferation of beneficial intestinal microbes, probiotics inhibit the growth of pathogenic bacteria to synergistically improve the integrity of the gut barrier (132). In a small placebo-controlled randomized control trial of only 35 patients with active CD, those who received a combination of prebiotic fructo-oligosaccharides/inulin and *Bifidobacterium longum* experienced significant improvements in histological samples after 3 and 6 months of the study. There was also a significant decrease in TNF-α expression at 3 months that was maintained through the rest of the study (118). Similar to studies related to probiotics and prebiotics, more large, high-quality trials are needed to determine the use and efficacy of synbiotics in CD.

### Therapeutic Dietary Modifications in CD

Diet is a major lifestyle factor that is significantly linked to the composition/function of the gut microbiota. In an analysis of genotype and microbiome data of healthy individuals, at least 20% of β-diversity is related to environmental factors, such as diet, drugs, and anthropometric measurements (102). In a five-day *ad libitum* dietary intervention, significant alteration in the gut microbiome composition and activity was evident in just 24 h of diet initiation (149). Those consuming an animal-based diet including meat, cheese, and eggs showed an increase in bile-tolerant microbes, such as *Alistipes, Bilophila*, and *Bacteroides*. In particular, the increase in *Bilophila wadsworthia* supports the association between dietary fat, increased bile acids secretion, and the expansion of microorganisms implicated in IBD. Furthermore, a reduction in the levels of Firmicutes, such as *Roseburia, Eubacterium rectale*, and *Ruminococcus bromii*, was associated with an animal-based diet (149). Analysis of fecal SCFAs suggested that changes in macronutrients in both diets resulted in a change in microbial metabolic activity. Animal-based diets demonstrated significantly high levels of amino acid fermentation and lower levels of products of carbohydrate fermentation (149). Branched chain SCFA that are products of amino acid fermentation were positively associated with putrefactive bacteria such as *Alistipes putredinis* and *Bacteroide*s spp. Meanwhile, products of carbohydrate fermentation, more abundant in the plant-based diet, were associated with clusters of saccharolytic microbes such as *Roseburia* spp., *E. rectale*, and *F. prausnitzii* (149).

Diet may be more relevant and have a stronger effect on CD than UC since most prior epidemiological studies on dietary risk factors have identified association with CD, but not UC (150–152). There also seems to be a slight difference in dysbiosis in different phenotypes of CD. A greater reduction in butyrate producers such as *F. prauznitzii* and increased abundance of *E. coli* is seen in ileal CD as compared to colonic CD, suggesting that there might be a stronger link between diet, microbiome, and CD phenotype (21). A plethora of studies have explored dietary patterns associated with CD. Many studies such as the Healthy Lifestyle in Europe by Nutrition in Adolescence Study and the prospective cohorts of the Nurses' Health Study (NHS) and Nurses' Health Study II (NHSII) have shown that a higher intake of fiber, specifically cruciferous vegetables and cereals, is associated with lower incidence of CD (150, 151). On the other hand, a prospective study of NHS I and NHSII demonstrated a reduction in CD risk with high dietary fiber, specifically derived from fruits (150).

The EPIC-IBD study found no evidence that dietary fiber was associated with CD or UC. However, a higher intake of cereal fiber in non-smokers was inversely associated with odds of developing CD (151). During analysis of 3 ongoing prospective cohort studies (NHS, NHSII, and The Health Professionals Follow-up Study (HPFS)), it was demonstrated that dietary patterns with high inflammatory potential were associated with increased risk for CD, but not UC. There seemed to be a dynamic risk with CD; not only was there a long-term pro-inflammatory diet associated with a higher risk, but also an increase in disease development from low to high inflammatory potential diet. In this study, the association between an inflammatory diet and the risk of CD remained unchanged for additional fiber intake. These results, confirmed by others (153), suggest that dietary fiber and low inflammatory potential foods are both important components of the diet to reduce CD risk (154). Examples of a high inflammatory potential diet is the Western diet, which is high in saturated fat, added sugar, and low in fiber (155). Khalili et al., showed in two large prospective studies in Sweden that poor adherence to the Mediterranean diet (a low-inflammatory potential diet) conferred a population attributable risk of 12% for later-onset Crohn's disease—suggesting that greater adherence to a Mediterranean diet is associated with a lower risk of later-onset CD (152). Moreover, Bolte et al., found that processed foods and animal-derived sources were associated with a higher abundance of microbes associated with inflammation such as Firmicutes, *Ruminococcus* spp., and endotoxin synthesis pathways (153). Meanwhile, plant-derived sources were associated with SCFA-producing bacteria and improved metabolic pathways (153). Table 3 includes various diets and their association with inflammation and risk of CD. These studies suggest that adherence to a low-inflammatory potential diet (i.e., Mediterranean diet) and the addition of fermentable dietary fiber may aid in reducing the risk of CD in those that are genetically predisposed.

**Table 3:** Dietary patterns associated with inflammation or increased incidence of CD.

**Study Type**	**Participants (*n*)**	**Duration**	**Outcome**	**Reference**
Observational	170 776	26 years	Long-term intake of dietary fiber, especially fruit, is associated with lower risk of CD but not UC	(150)
Prospective cohort	401 326		No associations between fiber from specific sources and risk of UC/CD	(151)
Prospective cohort	83 147	17 years (SD±5)	Mediterranean Diet associated with lower risk of CD	(152)
Prospective cohort	208 834 (NHS, NHSII, HPFS)	-	High dietary inflammatory potential associated with 51% higher risk of CD	(154)
Cross-sectional	1425 (CD, UC, IBS, HC)	-	Processed foods and animal- based foods associated with increased abundances of Firmicutes, *Ruminococcus* spp. Plant-based foods and fish positively associated with short- chain fatty acid-producing commensal bacteria	(153)

*CD, Crohn's disease; UC, Ulcerative colitis; SD, standard deviation; NHS, Nurses' Health Study (NHS); NHSII, Nurses' Health Study; HPFS, The Health Professionals Follow-up Study; IBS, Irritable Bowel Syndrome; HC, healthy controls*.

Therapeutic diets have the potential to both induce and maintain remission. Exclusive Enteral Nutrition (EEN) is the use of a liquid elemental or polymeric formula consumed exclusively for up to 12 weeks (156). EEN has shown remarkable success in inducing remission (157, 158) and is especially recommended as first-line therapy to induce remission in pediatric patients with CD (159). The mechanisms of EEN are related to its role in reducing antigens in whole foods, improving micronutrient and macronutrient deficiencies, and improving gut dysbiosis (158, 160, 161). EEN reduces microbiota diversity and initiates modulation of intestinal bacterial communities (156, 162). Notably, EEN reduces inflammation through the modulation of *Bacteroides* species. A significant positive correlation between a change in *Bacteroides-Prevotella* groups and a reduction in pediatric Crohn's disease activity index (PCDAI) (156, 162). Following EEN's success, many dietary therapies have focused on eliminating certain dietary components to improve disease severity. The Specific Carbohydrate Diet (SCD) consists of eliminating all grains, sugars (except for honey), milk products (except for hard cheeses and fermented yogurt), and most processed foods (163). This diet has shown some efficacy in inducing remission; however, long-term adherence is difficult due to the limitations of this diet. Therefore, diets offering a greater variety of foods, such as the modified-Specific Carbohydrate Diet (mSCD) and Mediterranean diet (MD), have been explored as an alternative means of dietary intervention. Both the mSCD and MD show comparable results to the SCD but boast a more liberal dietary pattern that is customizable and easier to follow. Studies involving the Mediterranean diet have shown promise regarding inducing and maintaining remission, improving inflammatory biomarkers, and quality of life (QoL). The Crohn's Disease Exclusion Diet and Partial Enteral Nutrition (CDED + PEN) approach has been explored and has shown to induce and sustain remission in 75% of pediatric CD patients at 12 weeks (164, 165). The CDED consists of the removal of animal fat, wheat, dairy, red meat, emulsifier, maltodextrin, inulin, and carrageenan and the addition of fruits and vegetables. CDED + PEN was associated with a reduction in Proteobacteria and intestinal permeability, as measured by a Lactulose/Mannitol Test (165). In addition, this diet-induced a decrease in abundance in *Haemophilus, Veillonella, Anaerostipes*, and *Prevotella*, and an increase in *Roseburia* and *Oscillibacter* (165). As shown in Table 4, plant-based and low inflammatory diets contribute to an improved microbiome profile along with disease amelioration.

**Table 4:** Dietary patterns to induce or maintain remission in Crohn's disease.

**Diet**	**Study type**	**Participants** **(*n*)**	**Duration (months)**	**Outcome**	**Reference**
**Active Crohn's disease**					
Mediterranean Diet	Open-label intervention	142 Adult	6	Improvement in BMI, waist circumference, liver steatosis, disease severity, inflammatory biomarkers, and quality of life	(166)
PREDIMED Mediterranean diet score	Observational	66 Adult	3	Daily intake of leafy green vegetables associated with FCP ≤ 100μg Higher omega 6:3 ratio associated with CRP ≤ 5 mg	(167)
Specific Carbohydrate Diet vs. Mediterranean Diet	RCT	194 Adult	3	Specific Carbohydrate Diet was not superior to the Mediterranean diet to achieve symptomatic remission, FCP response, and CRP response.	(168)
Specific Carbohydrate Diet vs. Modified Specific Carbohydrate Diet vs. Whole foods	RCT	18 Pediatric	3	All 3 diets were associated with high and comparable rates of clinical remission, and all had improvement in inflammation to differing degrees	(169)
Crohn's Disease Exclusion Diet and Partial Enteral Nutrition vs Exclusive Enteral Nutrition	RCT	74 Pediatric	3	CDED + PEN is better tolerated than EEN, both are effective at achieving remission in the short-term	(164, 165)
**Inactive Crohn's disease**					
Semi-vegetarian diet	Open-label intervention	22 Adult	24	SVD prevented relapse	(170)
High-meat vs. Low-meat	Observational	213 Adult	~11	Red/processed meat is not associated with time to relapse	(171)
Low FODMAP	RCT	52 Adult	1	Low FODMAP diet reduced gut symptoms scores and significantly lower abundance of *Bifidobacterium adolescentis, B. longum*, and *Faecalibacterium. prausnitzii*, no change in microbiome diversity and markers of inflammation	(172)

*RCT, randomized control trial; BMI, body mass index; FCP, fecal calprotectin; CRP, C-reactive protein; CDED + PEN, Crohn's Disease Exclusion Diet and Partial Enteral Nutrition; ENN, Exclusive Enteral Nutrition; SVD, Semi-vegetarian diet; Low FODMAP, Low fermentable oligosaccharides, disaccharides, monosaccharides, and polyols*.

### Fecal Microbiota Transplantation in CD

Fecal microbiota transplantation (FMT) is the transfer of fecal matter from a healthy donor to a person with dysbiotic gut microflora. The aim of FMT is the restoration of a healthy microbiota (173). FMT has been remarkably successful in the treatment of *Clostridioides difficle* infections (CDi) and is being explored as a therapeutic option for IBD; however, the results have not been as promising. Certain randomized control trials, such as a landmark FMT study by Moayeddi et al., have shown success in inducing clinical remission in patients with UC (174). In this study, success in remission induction was associated with a recent UC diagnosis, as the perturbation in microbial structure was easier to change in its early stages (174). The inconsistency in FMT results is likely due to the more complex pathogenesis of IBD as compared to CDi. Similar to probiotics and prebiotics, data regarding FMT and IBD is limited and heterogeneous, making it difficult to determine its absolute effect on IBD disease activity.

In a promising 5-year Chinese study by Xiang et al., a step-up FMT strategy was used in 174 patients with CD. This 3-step strategy used integrative treatment consisting of a single or multiple FMT in conjunction with steroids, immunomodulators, and exclusive enteral nutrition. Improvements in abdominal pain, hematochezia, fever, and diarrhea were seen from 1-month post-FMT to the end-of-study at 3 years post-FMT. This study was the largest cohort of patients that underwent FMT and had a long-term follow-up. It is important to note that this study neither used a control group, nor assessed endoscopic biomarkers, quality of life, or microbial analysis (175). However, the optimal time between FMT doses was approximately 4 months, a similar time frame as in a previous study by Li et al. (176). However, it becomes evident that FMT on its own does not induce long-term remission. In a multivariate analysis, it was shown that degree of dysbiosis, longer-disease duration (>5 years), and severity (HBI > 8) was associated with poorer response to FMT (33, 176). FMT seems more likely to be successful in CD in early stages, in milder disease, when administered in multiple courses and conjunction to other treatment modalities. FMT has the potential to provide a profound improvement in specific patients with CD, but information regarding donor characteristics and time of administration (early vs. late disease course) has yet to be explored in a standardized and controlled manner. One major issue with the majority of these FMT studies is that they are underpowered, are often open-label, do not account for the healthy donor effect, and have a lack of reproducibility.

## Intestinal Permeability and Crohn's Disease

Intestinal permeability (IP) refers to the functional property of the intestinal mucosal barrier that controls the interactions between the gut and gut microbes. Normal intestinal permeability allows for the coexistence of microbial symbionts of the host while preventing luminal penetration of macromolecules and pathogens (177). The purpose of the intestinal barrier is to reduce contact between luminal microbial contents and the mucosal immune system (178). In healthy humans, it acts as a semi-permeable physical barrier allowing selective movement of nutrients while protecting the body from pathogenic invasion (179). An impaired intestinal barrier and increased IP, also known as “leaky gut,” has been the focus of research as it appears to be a defining factor in the pathogenesis of IBD (177, 179). Epithelial integrity is characterized by a 4–5-day turnover of cell shedding into the intestinal lumen at the surface and the proliferation of multipotent stem cells within the intestinal crypt to replace the loss of cells (180). Disruption of intestinal barrier turnover contributes to invasion of luminal antigens and intestinal inflammation as seen in ulcerative colitis (UC) and Crohn's disease (CD) (181, 182).

Intestinal epithelial cells (IECs) are mechanically attached by the junctional complexes of tight junctions (TJs), adherence junctions, and desmosomes (183–185). These structures also control the paracellular transport of ions and small molecules between adjacent cells *via* passive transport. Patients with IBD display several TJ abnormalities leading to increased paracellular transport (186, 187). Patients with IBD display reduced expression and redistribution of TJs and their constituents such as occludins, claudins, and junctional adhesion molecules (JAM) (181, 182, 188, 189). Tumor-necrosis factor-α (TNF-α), a pro-inflammatory cytokine implicated in the progression of IBD, has been shown to modulate the transcription of TJ proteins (190, 191). Not only does TNF-α increase IP, but it also increases the rate of shedding of enterocytes *via* apoptosis which results in a lag of TJ redistribution to adhere cells together (181, 192).

Paracellular movement of molecules is limited by the function of TJs between IECs. Therefore, regulation of TJ function is essential for the normal movement of solutes between cells. Another factor affecting paracellular transport is epithelial damage through erosion or ulceration (193). Zonulin is a protein in humans that has been identified as a reversible regulator of TJ function. Zonulin modulates permeability by TJs disassembly leading to increased intestinal permeability (194). In autoimmune conditions, such as IBD, Celiac Disease, and Type 1 Diabetes, enhanced expression of zonulin has been observed, making it a biomarker of impaired gut function along with a potential target for therapy. Other biomarkers of intestinal permeability include glucagon-like peptide-2 (GLP-2). GLP-2 is involved in intestinal cell proliferation in the crypts of IECs, therefore beneficial in reducing the permeability of the gut (193, 194).

## Role of Microflora-Altering Therapy to Prevent Crohn's Disease

It is well-documented that there is a genetic component to CD susceptibility with up to 12% of patients having a family history of IBD. Genome-wide association studies (GWAS) have identified 240 single nucleotide polymorphisms (SNPs) in IBD (195). However, genetics alone do not explain the onset of CD, as many people with the identified alleles do not develop the disease (196). In a prospective study, Turpin et al. measured intestinal permeability by the urinary fractional excretion of lactulose-to-mannitol ratio (LMR) in 1,420 asymptomatic first-degree relatives (FDR) of patients with CD with a median follow-up of 7.8 years. An abnormal LMR (>0.3) was associated with a diagnosis of CD onset during the follow-up period, whereas the test was performed more than 3 years before the diagnosis of CD. Not only did these results demonstrate that increased intestinal permeability is associated with the risk of development of CD, but they also support the hypothesis that abnormal intestinal barrier function can contribute to the pathogenesis of CD and can serve as a biomarker for the risk of CD onset in healthy asymptomatic FDR (197). Another analysis by Turpin et al. of a GWAS of LMR within the same study population showed that host genetics provide only a small contribution to an abnormal LMR in FDR of patients with CD, suggesting that an abnormal LMR may be more likely a result of environmental triggers or insult (198). Morkl et al. found that intestinal permeability in women, as measured by serum zonulin, was reflective of diet composition including calories, protein, carbohydrate, sodium, and vitamin B_12_ intake. It also was associated with the composition of the gut microbiota. Specifically, butyrate-producing *Faecalibacterium* and *Ruminococcaceae* were significantly more abundant in the low-zonulin group (199). These results suggest that controlling environmental factors using diet and dietary supplements may affect abnormal LMR to improve intestinal permeability. Currently, there are no therapies approved by US Food and Drug Administration or Health Canada to directly target and restore the abnormal intestinal barrier. The use of prebiotics and a low-inflammatory diet could be promising as therapeutic agents to restore a defective mucosal barrier and reduce intestinal permeability, either directly and/or by restoring gut dysbiosis (177, 200).

## Conclusion

Approaches to modulate the gut microbiota in CD toward a healthier state are a topic of great interest. Despite the great advances in the gut microbiota field, we are far from fully uncovering the interrelations between the different bacteria, fungi, archaea, and viruses and how this is translated into host health. Current findings imply environmental factors, such as the Western diet, and some medications that can modulate directly or indirectly (through increased intestinal permeability) the intestinal microbiome, hence enabling the initiation of a cascade of pathophysiological changes. Concurrently, medicine regulatory authorities and drug developers should extend their pharmacodynamics and pharmacovigilance guidelines to incorporate possible drug-microbiota interactions with respect to safety and mode of action. In contrast, some diets, specifically Mediterranean-like diets, are suggested to restore eubiosis of the gut microbiome. Complementing a diet with other modulatory approaches, such as the addition of immune-modulating probiotics and prebiotics may improve the clinical efficacy and suppress chronic inflammation. Similarly, FMT has the potential to induce profound changes in the global gut community, thus rigorous safety and technical implementations are a prerequisite for a successful application in CD. Given the complexity of CD, it is crucial that future research focuses on the nuances of personalized medicine to recommend individually tailored care plans regarding therapeutics and nutraceuticals to prevent or delay the onset of Crohn's disease or reduce disease severity. Such knowledge in this rapidly evolving field is also important for clinicians and translational scientists to incorporate these microbiota-altering adjunct therapies into CD treatment options, along with awareness of the limitations of these therapies at this time, until more research is performed.

## Author Contributions

RR, RV, and LD conceptualized the manuscript. RR and RV wrote the manuscript. LD edited the manuscript. All authors contributed to the article and approved the submitted version.

## Funding

RR is funded by an educational grant from Janssen, Canada. RV and LD were funded by Weston Family Foundation Microbiome Initiative (Grant no. POP 2019) and Crohn's and Colitis Foundation of America.

## Conflict of Interest

The authors declare that the research was conducted in the absence of any commercial or financial relationships that could be construed as a potential conflict of interest.

## Publisher's Note

All claims expressed in this article are solely those of the authors and do not necessarily represent those of their affiliated organizations, or those of the publisher, the editors and the reviewers. Any product that may be evaluated in this article, or claim that may be made by its manufacturer, is not guaranteed or endorsed by the publisher.
